# Investigation of 11p15.5 Methylation Defects Associated with Beckwith-Wiedemann Spectrum and Embryonic Tumor Risk in Lateralized Overgrowth Patients

**DOI:** 10.3390/cancers15061872

**Published:** 2023-03-21

**Authors:** Beyhan Tüysüz, Serdar Bozlak, Dilek Uludağ Alkaya, Süheyla Ocak, Büşra Kasap, Evrim Sunamak Çifçi, Ali Seker, Ilhan Avni Bayhan, Hilmi Apak

**Affiliations:** 1 Department of Pediatric Genetics, Cerrahpasa Medical Faculty, Istanbul University-Cerrahpasa, 34098 Istanbul, Turkey; 2 Department of Pediatric Hematology and Oncology, Cerrahpasa Medical Faculty, Istanbul University-Cerrahpasa, 34098 Istanbul, Turkey; 3 Department of Orthopedics and Traumatology, Cerrahpasa Medical Faculty, Istanbul University-Cerrahpasa, 34098 Istanbul, Turkey; 4 Department of Orthopedics and Traumatology, Baltalimani Bone Diseases Training and Research Center, University of Health Sciences, 34470 Istanbul, Turkey

**Keywords:** 11p15 methylation defects, Beckwith–Wiedemann spectrum, lateralized overgrowth, Wilms tumor, embryonal tumors

## Abstract

**Simple Summary:**

Lateralized overgrowth may be isolated or accompany syndromes. Recently, international BWS consensus proposed that the patients with isolated lateralized overgrowth and epigenetic change related to this syndrome should be evaluated within the Beckwith–Wiedemann spectrum. The risk of cancer is high in patients with lateralized overgrowth, some patients also may be admitted presenting with a tumor. The aim of this study was to classify patients with lateralized overgrowth into isolated, atypical, and classic phenotypes according to consensus scoring, to investigate epigenetic alterations on chromosome 11p15.5, and to raise awareness for early detection and prevention of cancer.

**Abstract:**

The Beckwith–Wiedemann spectrum (BWSp) ranges from isolated lateralized overgrowth (ILO) to classic phenotypes. In this broad clinical spectrum, an epigenetic alteration on chromosome 11p15.5 can be detected. The risk for embryonal tumors is high, especially in patients with lateralized overgrowth (LO). The aim of this study is to investigate epigenetic alterations in 11p15.5 and tumor risk in 87 children with LO. The methylation level of 11p15.5 was examined in the blood of all patients and in skin samples or buccal swabs from 40 patients with negative blood tests; 63.2% of patients were compatible with the ILO phenotype, 18.4% were atypical, and 18.4% were classic. The molecular diagnosis rate was 81.2% for the atypical and classic phenotypes, and 10.9% for the ILO phenotype. In patients with epigenetic alterations, LO was statistically significantly more severe than in test negatives. Tumors developed in six (6.9%) of the total 87 patients with LO; four belonged to the atypical or classical phenotype (12.5%) and two to ILO (3.5%). Three of the four patients with atypical/classical phenotypes had pUPD11, one had IC1-GOM alteration, and two ILO patients were negative. We conclude that LO patients should be monitored for tumor risk even if their epigenetic tests are negative.

## 1. Introduction

Lateralized overgrowth (LO), also known as hemihypertrophy, is defined as an asymmetric regional overgrowth of the body and can be associated with many syndromes. The underlying causes may be genetic or epigenetic defects that lead to impaired cell growth and proliferation [1]. Isolated LO (ILO, OMIM #235000) is defined as a lateralized overgrowth in the absence of any other known malformation or dysplasia. Some overgrowth syndromes characterized by lateralized overgrowth have been associated with an increased risk of developing tumor, such as Beckwith–Wiedemann syndrome (BWS), PIK3CA-associated overgrowth syndrome, Proteus syndrome, and PTEN hamartoma tumor syndrome [2]. In BWS, there is a predisposition to embryonic tumors in early childhood caused by defects in a group of imprinted genes on chromosome 11p15.5 regulated by the imprinting control (IC) domains 1 and 2 [3]. Recently, the international BWS consensus proposed that ILO patients with BWS-related molecular findings should be evaluated under the Beckwith–Wiedemann spectrum (BWSp) according to their new clinical scoring system [4]. The BWSp ranges from ILO to atypical to classical phenotype; epigenetic alterations in the 11p15.5 domain can be detected in approximately 80–85% of cases [4,5]; 50% of patients have methylation loss in the IC2 domain (IC2-LoM), 20% have paternal uniparental disomy (pUPD11), and 5–10% have methylation gain in the IC1 domain (IC1-GoM) in the 11p15.5 region [5,6,7].

Duffy et al. [5] studied 322 patients according to the new BWS classification and found that 64.3% of patients had a classical phenotype, 17.1% had an atypical phenotype, and 18.6% had an ILO phenotype. Although LO is the main feature of ILO patients, it also occurs in the classic phenotype and more frequently in the atypical phenotype. It may be associated with any molecular subtype, although it is most common in patients with pUPD11 (60–85%) [7,8,9,10]. BWSp is a disease with mosaicism; some patients are diagnosed molecularly by secondary tissue testing. In one study, various tissue samples from BWSp’ affected individuals showed a prevalence of 43.4% for mosaicism [11].

The fact that embryonal tumor risk is higher in BWSp patients, especially in LO, has increased the importance of both clinical diagnosis and the detection of methylation alterations in the 11p15.5 region in this group of patients [12,13,14]. On the other hand, Wilms tumor (WT) and other embryonal cancers associated with BWSp may be the first recognized manifestation of the syndrome. MacFarland et al. [15] screened WT patients for 11p15 methylation changes and found 11p15 changes in eight of 12 WT cases with clinical BWS manifestations. As a result of these studies, it was emphasized that epigenetic testing related to BWS should be performed in larger groups of patients presenting with both LO and tumors.

The aim of this study is to investigate 11p15.5 methylation in both blood and skin samples or buccal swabs and to evaluate the frequency of embryonal tumors in patients with LO.

## 2. Materials and Methods

### 2.1. Patients

A total of 87 children were enrolled in this study. A clinical examination was performed by an experienced clinical geneticist. Birth and current weight, length, and head circumference of the patients and the height of the parents were recorded. All children with a length and/or circumference difference of ≥1 cm on one or more extremities compared with the contralateral side were accepted as LO. The length and circumference of the affected segment and the contralateral segment and the difference (in cm) were recorded. Patients with >2 cm difference in diameter and/or length between both extremities were classified as severe LO, and if the difference was between 1 and 2 cm, as mild LO. Patients’ characteristics were divided into two groups according to the BWSp scoring system: cardinal and suggestive. Cardinal features included macroglossia, omphalocele, lateralized overgrowth, multifocal WT or nephroblastomatosis, hyperinsulinism, and pathologic findings. The suggestive features were birth weight greater than >2 standard deviations (SD), facial nevus simplex, polyhydramnios, placentamegaly, ear crease/pit, transient hypoglycemia, organomegaly, umbilical hernia, diastasis recti, and typical embryonal tumors [4]. Two points were assigned for each cardinal feature and one point was assigned for each suggestive feature. The patients with a clinical score ≥6 points with two or more cardinal features (except hyperinsulinism and lateralized overgrowth) were classified as classic BWSp. Patients with a clinical score <6 points with at least one cardinal feature; or patients with a clinical score ≥6 points with hyperinsulinism and lateralized overgrowth as two cardinal features; or patients diagnosed with BWS after presenting with hyperinsulinism or the tumor (except for a single cardinal feature LO) were classified as atypical BWSp. Patients with a clinical score <4 with LO as the only cardinal feature were classified as ILO group [5]. Written informed consent was obtained from the patients’ parents/guardians for all types of samples.

### 2.2. Genetic Test Algorithm

In the first stage, Methylation-specific multiplex ligation-dependent probe amplification (MS-MLPA) (MRC Holland, Amsterdam, The Netherland) analysis was applied for chromosomes 11p15.5 domain in all patients from blood samples (Figure S1). Skin biopsy samples were taken from the side with hemihypertrophy in 13 patients from who parental consent could be obtained and buccal swabs from 27 patients with normal blood epigenetic tests. In total, a second tissue sample was obtained from 40 of 56 patients.

### 2.3. MS-MLPA

The methylation level of the domain of chromosome 11p15.5 and the presence of UPD were detected using MS-MLPA, Salsa ME-030-C3 BWS/RSS with methylation-sensitive probes for regions H19/IGF2: IG-DMR (IC1) and KCNQ1OT1/TSS-DMR 1(IC2). This kit contained four MS-MLPA probes for the H19 gene and four MS-MLPA probes for the KCNQ1OT1 locus.

In this study, the methylation levels for 11p15.5 obtained in our laboratory by the same method in 30 healthy children were used as a control group. The methylation indices for ICR2 were mean ± SD: 0.53 ± 0.049 (range 0.41–0.64), whereas the mean ± SD for ICR1 was 0.53 ± 0.046 (range 0.41–0.62). A value between 0 and 0.484 in the IC2 region was accepted as methylation loss (LoM) and a value between 0.576 and 1 in the ICR1 region was accepted as methylation gain (GoM). The presence of both GoM in ICR1 and LoM in ICR2 was scored as paternal uniparental disomy of 11p15.5. Since the methylation indices in the patients with paternal 11p15.5 UPD were between normal and threshold values, with a range of 0.61–0.69 (0.66 ± 0.035) for ICR1 and 0.28–0.47 (mean ± SD: 0.38 ± 0.07) for ICR2, they were accepted as borderline UPD [16].

We also examined the methylation status in other imprinted loci in the patients with ICR2-LOM. We used the MS-MLPA assays ME031-B2 GNAS to examine imprinted loci on chromosome 20 (contains probes for the GNAS locus) and ME032-A1 UPD7-UPD14 to examine imprinted loci on chromosomes 6, 7, and 14 (contains probes for PLAGL1 on chromosome 6, GRB10 and MEST on chromosome 7, and DLK1, MEG3, MIR380, and RTL1 on chromosome 14). Unfortunately, GWpUPD could not be performed in this study because the frequency of GWpUPD in patients with pUPD is low (1–2%) and can be detected in patients and parents by SNP array analysis.

### 2.4. Statistical Methods

Statistical analyses were performed using IBM Corp. SPSS Statistics^®^ version 21.0. The chi-square test and Fisher’s exact test were used to determine whether the difference between the two groups was significant. A probability value (*p*-value) of less than 0.05 was considered statistically significant.

## 3. Results

This cohort included 85 patients with LO and two patients with LO who presented with embryonal tumors. Forty-eight of our patients were male, and 39 were female. The median age at admission was 18 months (range 1–185 months) and the median follow-up was 48 months (range 3–360 months).

### 3.1. Clinical Characteristic

**Figure 1:**
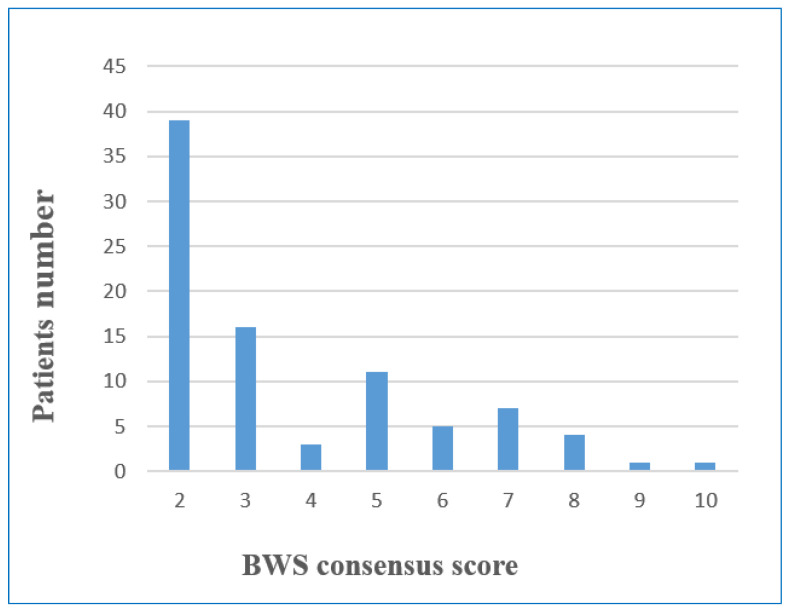
Distribution of patient numbers according to BWSp consensus score.

The cardinal and suggestive findings and the classification of all patients according to the BWS consensus are shown in Table S1. The patients with a score of 2 or 3 according to the BWSp scoring system were classified as ILO, and those with a score of >4 were classified as atypical or classic phenotype according to the international BWS consensus criteria. In the clinical evaluation of 87 patients with LO, 55 (60.9%) of the patients were compatible with ILO, and 32 (39.1%) were compatible with the atypical or classic phenotype (Figure 1).

The common clinical features accompanying LO were macroglossia (*n* = 26; 29.8%), ear crease/pit (*n* = 17; 19.5%), umbilical hernia/diastasis recti (*n* = 14; 16%), organomegaly (*n* = 13; 14.9%), transient hypoglycemia (*n* = 11; 12.9%), facial nevus simplex (*n* = 8; 9.1%), omphalocele (*n* = 7; 8%) and typical BWSp tumors (*n* = 7; 8%) (Table S1).

When the LO localization was evaluated, 56.4% had both lower and upper extremities; 40.3% right, 16.1% left, and 3.4% contralateral. 26.4% of patients had an isolated lower right side, 10.6% had an isolated lower left side, 2.3% had an isolated upper left side, and 1.2% had an isolated upper right side. When patients were classified according to the severity of LO involvement, 58.4% were of the mild type, and 41.4% were of the severe type (Table 1).

### 3.2. Molecular Results

In the blood samples of 31 of the 87 patients (35.6%) with LO, a methylation defect was detected in the 11p15.5 region. Second samples were obtained from 40 of 56 patients with negative blood tests, skin biopsies from 13 patients, and buccal swabs from 27 patients. Methylation analyses of region 11p15.5 performed in these samples revealed epigenetic alterations in only one patient.

The overall diagnostic rate was 35.6%; this rate was 10.9% in the ILO and 81.25% in the atypical/classical group. As a result of analysis of the 11p15.5 region by MLPA-MS, IC1-GOM, IC2-LOM, and pUPD11 were identified in 2.3%, 19.5%, and 14.9%, respectively (Table 2). In addition, we examined methylation status at other imprinted loci in patients with ICR2-LOM. No abnormal methylation status was detected in the imprinted regions on chromosomes 6, 7, 14, and 20.

**Figure 2:**
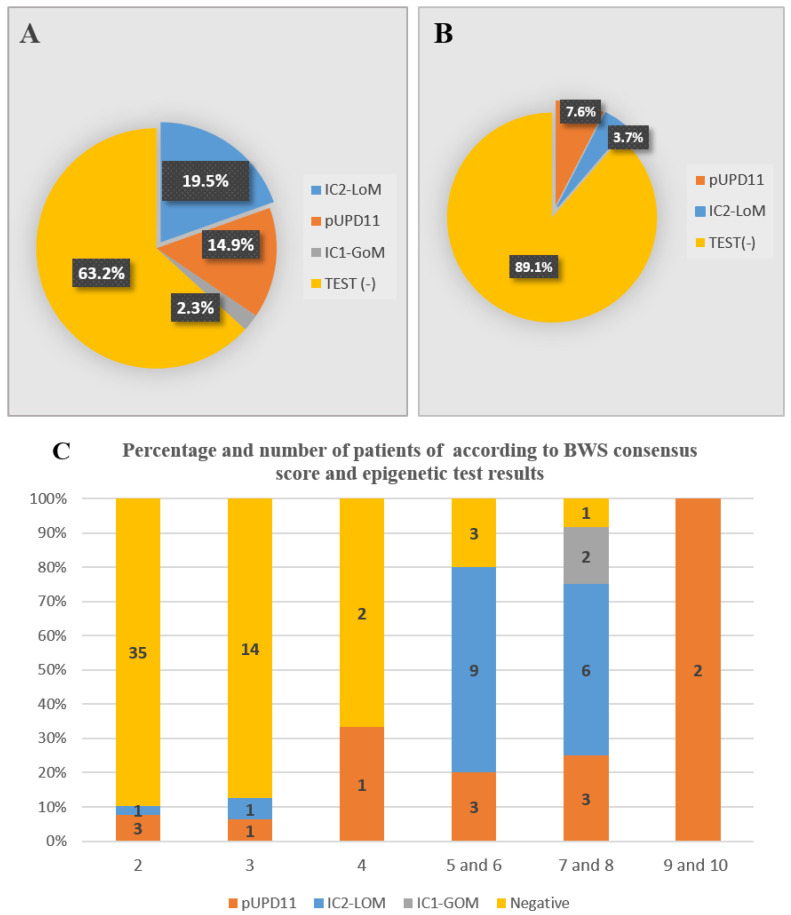
Epigenetic alterations in all patients with lateral overgrowth (**A**) and isolated lateralized overgrowth (**B**). The distribution of epigenetic alterations according to clinical scoring in LO patients (**C**).

The distribution of epigenetic changes in all patients and ILO subgroups are shown in Table 2 and Figure 2A,B. Among the patients with atypical/classical phenotype; 6.25% had IC1-GOM, 46.9% IC2-LOM, 28.1% pUPD11, and 18.75% patients had a negative test. While the test was normal in 89.1% of ILO patients, pUPD11 was shown in four patients (7.6%) and IC2-LOM (3.7%) in two patients.

The frequency of molecular diagnosis according to clinical score is shown in Figure 2C; epigenetic alterations were detected in six ILO patients who had a score of 2 or 3 (10.9%); two of the 15 patients with a clinical score of 3, four of the 38 patients with a score of 2. While the diagnosis rate was 35% in atypical/classic patients with a score of 4, it was 80% in patients with a score of 5 and 6, 90% in patients with a score of 7 and 8, and 100% in patients with a score of 9 and 10.

Lateral overgrowth was severe in five of six patients with ILO who had epigenetic alterations and mild in 32 of 49 patients with negative tests, and the difference between the two groups was statistically significant (Table 3; *p* < 0.05). However, no significant association was found between test positivity and LO severity in the atypical/classical groups.

### 3.3. The Incidence of Embryonal Tumors

Eighty-five patients with LO were followed up to a median age of 6.1 years; WT developed in three, and adrenocortical carcinoma in one between 10.5 and 36 months. In the atypical/classical group, pUPD11 was detected in two of the three patients who developed tumors and IC1-GOM in one, whereas epigenetic testing was normal in one patient with ILOs who developed WT. Two patients with tumors as initial findings, and also had LO were scored 4 and 3, respectively; one belonged to the ILO group and the other to the atypical phenotype group. One of two patients with LO, who developed WT at 12 months of age, had a right-sided LO, and another patient who developed WT at 36 months of age had an isolated lower right LO. Tumors developed in six (6.9%) of the total 87 patients with LO. This rate was 3.8% in patients with ILO. The LO severity of these patients was compared with that of patients who did not have a tumor, and no significant difference was found (*p* > 0.05).

## 4. Discussion

The BWS spectrum (BWSp) consists of the classical phenotype, ILO, and the atypical group with 11p15.5 methylation defects that do not fit into these two categories [4]. With the development of BWSp, attention has been drawn to patients in the atypical and ILO groups who have a higher risk of cancer, and studies have been conducted to investigate these two groups in recent years. In our study, we classified 87 children with LO according to clinical subgroups of BWSp; 63.2% of the patients were compatible with ILO and 32.8% with atypical/classic phenotypes. We found that BWSp-related epigenetic changes occurred in 81.25% of patients with atypical and classical phenotypes and in 10.9% of patients with ILO. About 20% of clinically diagnosed patients with the BWSp spectrum do not have a genetic/epigenetic defect [4,5,17]. In one study, 16% of 94 patients with LO had epigenetic alterations in 11p15.5, while only three (6.25%) of 48 ILO patients in this group were affected [18]; of these three ILO patients, two had pUPD11 and one ICR2-LOM. In our study, ILO patients with positive epigenetic tests (10.9%); 7.6% of these had pUPD11 and 3.7% ICR2-LOM. Lateral overgrowth can occur in all molecular subtypes of BWSp; it is most commonly associated with pUPD11 [4,10,11,13]. In a BWS cohort of 1000 people, 57.3% of patients with hemihypertrophy had epigenetic changes in pUPD11 and 35.1% in ICR2-LOM [6]. One study found that 25% of 75 patients with ILO had epigenetic changes in the 11p15.5 region; of these, 15% had pUPD11 [19].

Radley et al. [18] compared their own results with Duffy’s study, in which an epigenetic diagnosis was made in 43.9% of ILO patients [5], and suggested that their lower genetic diagnosis rate was probably related to the use of other tissue samples in Duffy’s study and patient characteristics. In our study, we were able to epigenetically test samples from the second tissue in 40 of 56 patients whose blood test was negative and detected epigenetic alterations in only one patient. A second reason could be that awareness and demand for epigenetic testing have increased in recent years as ILO’ has been recognized as a manifestation of BWSp. Because patients in our previously published BWS cohort were diagnosed before BWS consensus scoring, there were no ILO patients with less than 4 points of BWS-related epigenetic 11p15.5 alterations [20]. Moreover, in the studies that showed a higher diagnosis rate based on the second tissue, BWS patients of the classic type formed the majority [15,16,21]. However, in both our study and the study by Radley et al. [18], which included patients with LO, the proportion of patients with ILO who had a negative blood test was high.

In the study presented here, the molecular diagnosis rate correlated positively with the clinical score according to the BWS scoring system; it was 80% in patients with 4 points, 90% in patients with 7 and 8 points, and 100% in patients with 9 and 10 points similar to the previous study [18].

Most of the patients presented here (59.8%) had LO in both the lower and upper extremities, with the right side more affected. This was followed by isolated hemihypertrophy of the right lower extremity (26.4%). When previous studies were examined, it was found that the right side was more affected in patients, but this finding was not mentioned in the studies [22,23,24]. In one study, BWSp patients were classified as mild when the difference was 1–2 cm and severe when it was more than 2 cm, depending on the severity of LO [22]; while LO had milder courses in isolated lateralized patients, more severe courses were observed in patients with BWSp over time, especially in patients with the molecular subtype pUPD11. When we examined the relationship between LO severity and molecular test positivity, six patients with ILO with epigenetic alterations at 11p15.5 had severe LO, whereas patients with negative epigenetic test had mostly mild LO, and the difference was statistically significant (*p* < 0.05). Four of the six patients had LO right-sided involvement, one had left-sided LO involvement, and the other had contralateral involvement. Three of the five patients with severe LO had epigenetic pUPD11 alterations. We found no correlation between molecular test positivity and severity in the atypical/classical group. The correlation between severity and involvement of the right side of LO and test positivity has not been reported previously.

The most common clinical features accompanying LO were macroglossia (29.8%), ear crease/pit (19.5%), umbilical hernia/diastasis recti (16%) and organomegaly (14.9%) in our study and these findings were consistent with previous studies (18, 25). LO is commonly associated with hyperinsulinism; Kalish et al. [20] studied 28 children with hyperinsulinism and BWS and found that 26 of them were associated with lateralized overgrowth and pUPD11.

BWS is a well-defined tumor predisposition syndrome, and 7.4–10% of patients develop embryonal tumors (WT, hepatoblastoma, neoblastoma, adrenal cell carcinoma) in childhood, usually before the age of 7 years [13,14,25,26,27,28,29]. Mussa et al. [25] studied the characteristics of 318 BWS patients and reported a significant correlation between malignant neoplasms and lateralized overgrowth. In our study, four of 85 patients with LO, who were followed for an average of 6.1 years, developed a tumor between 10.5 and 36 months; three WT’ and one adrenocortical carcinoma. Of the two patients in whom WT was the initial finding and who also had LO on examination, one was of the ILO phenotype and the other of the atypical phenotype; WT developed at 12 months in the first patient and at 36 months in the patient with the atypical phenotype. In three studies investigating the incidence of embryonal tumors in children with the ILO phenotype, the tumor incidence was 5.9%, 2.6%, and 1.2%, respectively [23,30,31]. Similar to these studies, two of 55 patients (3.4%) in our ILO group also developed tumors; molecular testing was negative in two ILO patients with embryonal tumors, while three of four patients with atypical and classic phenotypes had pUPD11 and an IC1-GOM. Among the molecular subgroups of BWSp patients, the risk of tumor development was highest in patients with IC1-GOM (28%), 2.5% in IC2-LOM, and 16% in pUPD11 [2,12,26]; 152 patients with LO were evaluated for tumor frequency; in 34 patients who developed tumors, epigenetic alterations were detected in 61.8% pUPD11, 32% IC1-GOM, and 5.9% IC2-LOM [14]. Another study reported that the molecular subgroup with the highest tumor incidence was pUPD in LO patients [32]. In our study, the molecular test was negative in two ILO patients with embryonal tumors, while three out of four patients with atypical and classic phenotypes had pUPD11 and an IC1-GOM.

Since BWSp is a mosaic disease, testing more than one tissue source (buccal swab, tongue, skin sample, etc.) increases the diagnostic yield [33]. The limitation of this study is that we could not obtain skin samples from the side with hemihypertrophy from all test-negative patients. Another limitation is that in the cases where we found pUPD11, GWpUPD could not be performed.

## 5. Conclusions

The clinical features of most of our patients (63.2%) with LO were consistent with the ILO subgroup; the remaining patients belonged to the atypical/classical phenotype. The molecular diagnosis rate in the atypical/classical groups was 81.25%, whereas it was 10.9% in the ILO group. The epigenetic alteration in 11p15.5 was ICR2-LOM in 46.9% of patients, 11pUPD in 28.1%, and ICR1-GOM in 6.2% of patients with atypical and classic phenotypes. However, in the ILO phenotype, 3.7% of patients had ICR2-LOM and 7.6% had 11pUPD. In most patients, the upper and lower sides of the body were affected together, usually the right side. LO was severe in 41.1% of patients and mild in 58.9%. LO was more severe and predominantly right-sided in ILO patients with epigenetic alterations. Tumors developed in six (6.9%) of all patients, whereas its incidence in the ILO group was 3.8%. Three of four patients with atypical/classical phenotype who developed tumors had three pUPD11 and one ICR1-GOM, while the test was negative in two patients with ILO. LO can sometimes be the only detectable phenotypic feature of BWSp. We recommend evaluating clinical findings, monitoring all cases of LO, and performing epigenetic testing in atypical and ILO cases that are at high risk for tumors, even in cases with a negative blood test.

## Figures and Tables

**Table 1:** Distribution of lateralized overgrowth by severity and location.

Localization	Severe (%)	Mild (%)	Total (%)
Right side (upper/lower limb)	15 (17.6%)	20 (23.6%)	35 (40.3%)
Left side (upper/lower limb)	4 (4.7%)	10 (11.8%)	14 (16.1%)
Contralateral (upper/lower limb)	2 (2.4%)	1 (1.1%)	3 (3.4%)
Isolated right lower limb	12 (13.8%)	11 (12.5%)	23 (26.4%)
Isolated left lower limb	3 (3.5%)	6 (7.05%)	9 (10.3%)
Isolated left upper limb	0	2 (2.3%)	2 (2.3%)
Isolated upper right limb	0	1 (1.2%)	1 (1.2%)
**Overall**	36 (41.4%)	51 (58.6%)	87 (100%)

**Table 2:** Epigenetic alterations in the patients with lateralized overgrowth according to clinical phenotype.

Clinical Phenotype	Epigenetic Change				Negative Testing	Total
	ICR1- GOM	ICR2- LOM	11p UPD	Total		
**Atypical and Classical**	2 (6.2%)	15 (46.9%)	9 (28.1%)	26 (81.25%)	6 (18.7%)	32 (100%)
**ILO**	-	2 (3.7%)	4 (7.6%)	6 (10.9%)	49 (89.1%)	55 (100%)
**Total**	2 (2.3%)	17 (19.5%)	13 (14.9%)	32 (36.8%)	55 (63.2%)	87 (100%)

ILO: Isolated lateralized overgrowth; IC1-GoM, Imprinting center 1 gain of methylation; ICR2-LoM, Imprinting center 2 loss of methylation; pUPD11, Paternal uniparental disomy of chromosome 11.

**Table 3:** The relationship between epigenetic alteration in region 11p15.5 and the severity of lateralized overgrowth.

Severity of LO	Epigenetic Change				Negative Testing	Total	*p* Value *
	IC1 GOM	IC2 LOM	11p UPD	Total			
**ILO group**							***p* = 0.033 < 0.05**
**Severe**	0	2	3	5	17	22	
**Mild**	0	0	1	1	32	33	
**Total**	0	2	4	6	49	55	
**Atypical/classical group Severe**	0	5	4	9	4	13	***p* = 0.194 > 0.05**
**Mild**	2	10	5	17	2	19	
**Total**	2	15	9	26	6	32	

LO: lateralized overgrowth; ILO: Isolated lateralized overgrowth; BWSp: Beckwith–Wiedemann syndrome spectrum; IC1-GoM, Imprinting Center 1 gain of methylation; ICR2-LoM, Imprinting Center 2 loss of methylation; pUPD11, Paternal uniparental disomy of chromosome 11; * Shows the statistical analysis between of the patients with epigenetic change and -negative testing.

## Data Availability

The data that support the findings of this study are available from the corresponding author upon reasonable request.

## Supplementary Materials

The following supporting information can be downloaded at: https://www.mdpi.com/article/10.3390/cancers15061872/s1, Figure S1: The diagnostic algorithms of the patients included in this study; Table S1: The cardinal and suggestive findings and scoring of all patients according to the BWS consensus, and moleculer results.
