# Supplementary material for: Investigation of 11p15.5 Methylation Defects Associated with Beckwith-Wiedemann Spectrum and Embryonic Tumor Risk in Lateralized Overgrowth Patients
Source: Cancers (Basel). 2023 Mar 21;15(6):1872. doi: 10.3390/cancers15061872 (PMC10046725; doi:10.3390/cancers15061872)
Supplement: Supplementary file 1 [file cancers-15-01872-s001.zip › cancers-2244247-supplementary.pdf]

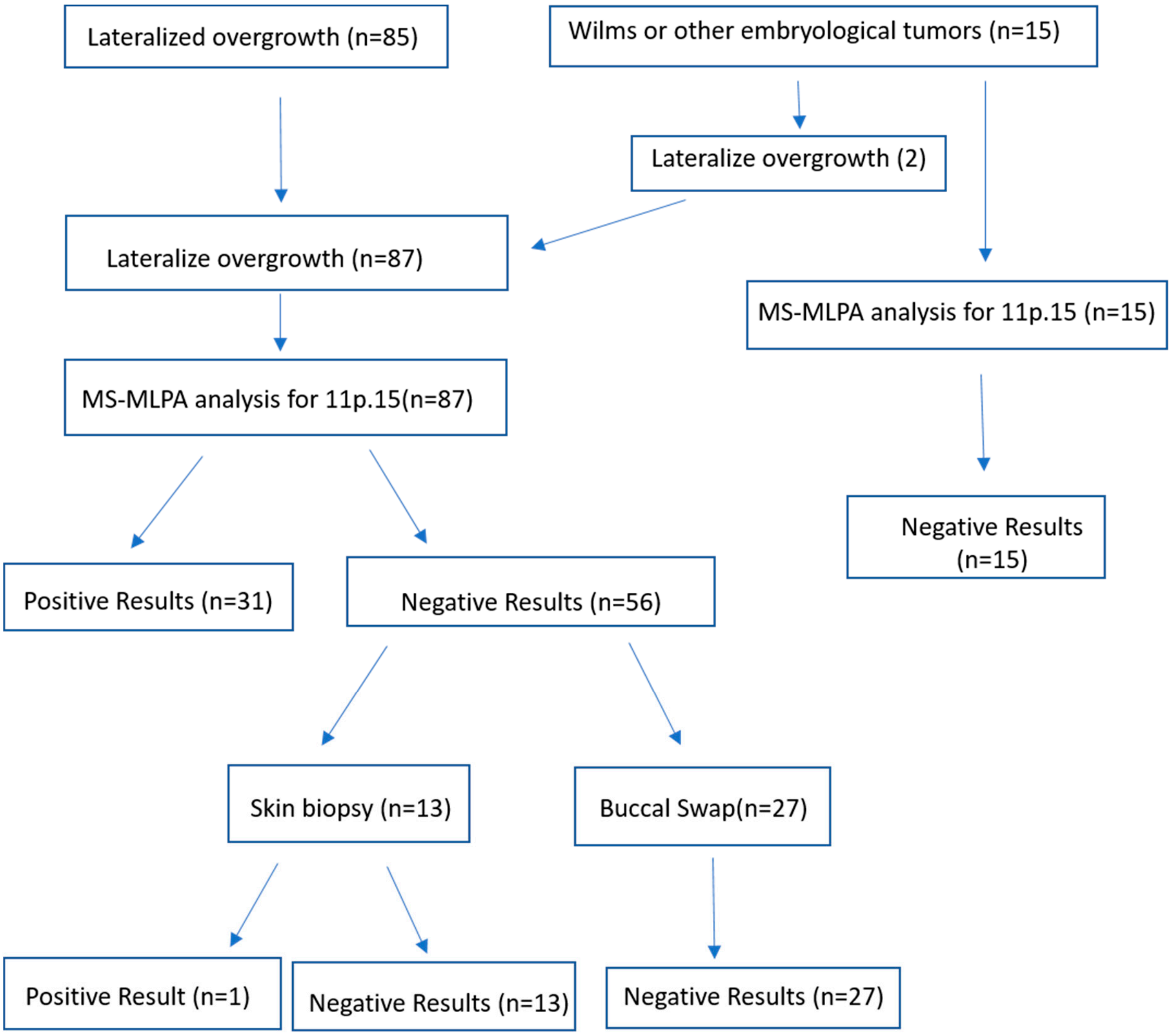

Figure S1. The diagnostic algorithms of the patients included in this study. MS-MLPA:

Abbreviation: MS-MLPA: Methylation-specific multiplex ligation-dependent probe amplification

Table S1. The cardinal and suggestive findings and scoring of all patients according to the BWS consensus, and moleculer results

Patient number	Diagnosis age /Gender	Cardinal and supportive features designated by the BWS consensus (Brioude et al., 2018)														Scorin g	BWS clinical spectrum	MS-MLPA for 11p15.5 region	
		Cardinal features (2 point)						Supportive features (1 point)											
		Pathologic	Multifocal/bilateral WT or nephroblastomatos	Macroglossia	Omphalocele	Lateralized overgrowth (severe or mild/localization)	Hyperinsulinism	Typical BWSp tumours	Birth weight greater>2SDS	Umbilical hernia/ diastasis recti	Ear crease/pit	Organomegaly	Facial nevus simplex	Polyhydramnios and	Transient hypoglycemia			blood sample	Skin/ buccal sample
1	7 mo/M	-	-	-	-	Severe/ right lower limb	-	-	-	+	-	-	-	-	-	3	ILO	pUPD11	-
2	5 yr/M	-	-	-	-	Severe/ contralateral	-	-	-	-	-	-	-	-	-	2	ILO	pUPD11	-
3	10 mo/F	-	-	-	-	Severe/ right lower limb	-	-	-	-	-	-	+	-	-	3	ILO	IC2-LoM	-
4	11 yr/F	-	-	+	+	Severe/ right upper and lower limb	-	-	+	+	+	-	-	-	-	9	Class ical	pUPD11	-
5	3 yr/M	-	-	+	-	Mild/ left upper and lower limb	-	-	-	+	+	+	-	-	-	7	Class ical	IC2-LoM	-
6	8 mo/M	-	-	+	-	Mild/ left lower limb	-	-	+	+	+	+	+	-	+	10	Class ical	pUPD11	-
7	5 yr/M	-	-	-	-	Severe/ right upper and lower limb)	-	-	-	-	-	-	-	-	-	2	ILO	IC2-LoM	-
8	3 mo/F	-	-	+	-	Mild/ right upper and lower limb	-	-	-	+	+	-	+	-	-	7	Class ical	IC2-LoM	-
9	7 mo/M	-	-	-	-	Severe/ left lower limb	-	-	-	-	-	-	-	-	-	2	ILO	pUPD11	-
10	4 yr/M	-	-	-	-	Mild/ right upper and lower limb	-	-	-	-	-	-	-	-	-	2	ILO	Borderlin e pUPD11	-
11	6 mo/M	-	-	-	-	Severe/ right upper and lower limb	-	-	-	+	+	+	-	-	-	5	Atypi cal	TEST (-)	IC2- LOM
12	9 yr/F	-	-	-	-	Severe/right lower limb	-	-	-	-	-	-	-	-	-	2	ILO	TEST (-)	TEST (-)
13	3 mo/M	-	-	-	-	Severe/ right upper and lower limb	-	-	-	-	-	-	-	-	-	2	ILO	TEST (-)	TEST (-)
14	8 yr/M	-	-	-	-	Severe/ left lower limb	-	-	-	-	-	-	-	-	-	2	ILO	TEST (-)	TEST (-)
15	6 mo/F	-	-	-	-	Mild/ right upper and lower limb	-	-	-	-	-	-	+	-	-	3	ILO	TEST (-)	TEST (-)
16	6 mo/M	-	-	-	-	Mild/ right upper and lower lim)	-	-	-	-	-	-	-	-	-	2	ILO	TEST (-)	TEST (-)
17	6 yr/M	-	-	-	-	Mild/ left upper and lower limb	-	-	-	-	-	-	-	-	-	2	ILO	TEST (-)	TEST (-)
18	2 yr/M	-	-	-	-	Mild/ left upper limb	-	-	-	-	-	-	-	-	-	2	ILO	TEST (-)	TEST (-)
19	16 yr/M	-	-	-	-	Severe/ right upper and lower limb)	-	-	-	-	-	-	-	-	-	2	ILO	TEST (-)	TEST (-)
20	6 mo/F	-	-	-	-	Severe/ right upper and lower limb	-	-	-	-	-	-	-	-	-	2	ILO	TEST (-)	TEST (-)

21	6 mo /M	-	-	-	-	Severe/ right upper and lower limb	-	-	-	+	-	-	-	-	-	3	ILO	TEST (-)	TEST (-)
22	4 yr/M	-	-	-	-	Mild/ right lower Limb	-	-	-	-	-	-	-	-	-	2	ILO	TEST (-)	TEST (-)
23	9 mo/F	-	-	-	-	Mild/ right upper and lower limb	-	-	+	-	-	-	-	-	-	3	ILO	TEST (-)	TEST (-)
24	9 mo/F	-	-	+	-	Severe/ left face and left lower limb	-	-	+	-	-	-	+	-	-	6	Atypical	TEST (-)	TEST (-)
25	3 yr/M	-	-	-	-	Severe/ left upper and lower limb	-	-	-	-	-	-	-	-	-	2	ILO	TEST (-)	TEST (-)
26	1 yr/M	-	-	-	-	Mild/ left upper and lower limb	-	-	-	-	-	-	-	-	-	2	ILO	TEST (-)	TEST (-)
27	4 mo/F	-	-	-	-	Severe/ right lower limb	-	-	-	-	+	-	-	-	-	3	ILO	TEST (-)	TEST (-)
28	1 mo/F	-	-	-	-	Mild/ right upper and lower limb	-	-	-	-	+	-	-	-	-	3	ILO	TEST (-)	TEST (-)
29	3 yr/M	-	-	-	-	Mild/ right lower limb	-	-	-	-	-	-	-	-	-	2	ILO	TEST (-)	TEST (-)
30	5 mo/M	-	-	-	-	Mild/ right upper and lower limb	-	-	-	-	-	-	+	-	-	3	ILO	TEST (-)	TEST (-)
31	8 yr/F	-	-	-	-	Mild/ right lower limb	-	+	-	-	-	-	-	-	-	3	ILO	TEST (-)	TEST (-)
32	7 yr/F	-	-	-	-	Severe/ right upper and lower limb	-	-	-	-	-	-	-	-	-	2	ILO	TEST (-)	TEST (-)
33	11 yr/M	-	-	-	-	Mild/ right upper and lower limb	-	-	-	-	-	-	-	-	-	2	ILO	TEST (-)	TEST (-)
34	6 mo/F	-	-	-	-	Mild/ right lower limb)	-	-	-	-	-	-	-	-	-	2	ILO	TEST (-)	TEST (-)
35	4 yr/F	-	-	-	-	Severe/ right upper and lower limb	-	-	-	-	-	-	-	-	-	2	ILO	TEST (-)	TEST (-)
36	6 yr/M	-	-	-	-	Mild/ right upper and lower limb	-	-	-	-	-	-	-	-	-	2	ILO	TEST (-)	TEST (-)
37	3 yr/M	-	-	-	-	Mild/ right lower limb	-	-	-	-	-	-	-	-	-	2	ILO	TEST (-)	TEST (-)
38	1 yr/F	-	-	-	-	Mild/ right lower limb	-	-	-	-	-	+	-	-	-	3	ILO	TEST (-)	TEST (-)
39	1 yr/F	-	-	-	-	Mild/ right upper limb	-	-	-	-	-	-	-	-	-	2	ILO	TEST (-)	TEST (-)
40	2 yr/M	-	-	-	-	Mild/ right lower limb	-	-	-	-	-	-	-	-	-	2	ILO	TEST (-)	TEST (-)
41	2 yr/F	-	-	-	-	Mild/ left upper and lower limb	-	-	-	+	-	-	-	-	-	3	ILO	TEST (-)	TEST (-)
42	6 mo/F	-	-	-	-	Mild/ right lower limb	-	-	-	-	-	-	+	-	-	3	ILO	TEST (-)	TEST (-)
43	2 yr/F	-	-	-	-	Severe/ right lower limb	-	-	-	-	-	-	-	-	-	2	ILO	TEST (-)	TEST (-)
44	3 yr /F	-	-	-	-	Severe/ right lower limb	-	-	-	-	-	-	-	-	-	2	ILO	TEST (-)	TEST (-)
45	11 yr/F	-	-	-	-	Mild/ sol lower limb	-	-	-	-	-	-	-	-	-	2	ILO	TEST (-)	TEST (-)
46	2 yr /M	-	-	-	-	Severe/ right lower limb	-	-	-	-	-	-	-	-	-	2	ILO	TEST (-)	-
47	5 mo/M	-	-	-	-	Mild/ right upper and lower limb	-	-	-	-	-	-	-	-	-	2	ILO	TEST (-)	-
48	2 yr/M	-	-	-	-	Severe/ right upper and lower limb	-	-	-	-	-	-	-	-	-	2	ILO	TEST (-)	-

49	1 yr /F	-	-	-	-	Mild/ left upper and lower limb	-	-	-	-	-	-	-	-	+	3	ILO	TEST (-)	-
50	3 yr /F	-	-	-	-	Severe/ right upper and lower limb	-	-	-	-	-	-	-	-	-	2	ILO	TEST (-)	TEST (-)
51	8 mo/F	-	-	-	-	Mild/ right upper and lower limb	-	-	-	-	-	-	-	-	-	2	ILO	TEST (-)	-
52	6 yr /F	-	-	-	-	Mild/Contralateral	-	-	-	-	-	-	-	-	-	2	ILO	TEST (-)	TEST (-)
53	10 yr/F	-	-	-	-	Severe/Contralateral	-	-	-	-	-	-	-	-	-	2	ILO	TEST (-)	TEST (-)
54	4 yr/F	-	-	-	-	Mild/Right lower limb	-	-	-	-	-	-	-	-	-	2	ILO	TEST (-)	TEST (-)
55	10 yr/F	-	-	+	-	Mild/left lower limb	-	-	-	+	-	-	-	-	-	5	Atypical	TEST (-)	-
56	2 yr/F	-	-	-	-	Mild/ left upper and lower limb	-	-	-	-	-	+	-	-	-	3	ILO	TEST (-)	-
57	9 yr/F	-	-	-	-	Mild/ left lower limb	-	-	-	-	-	-	-	-	-	2	ILO	TEST (-)	-
58	4 yr/M	-	-	-	-	Mild/ right lower limb	-	-	-	-	-	-	-	-	-	2	ILO	TEST (-)	-
59	1 yr/M	-	-	-	-	Severe/ right lower limb	-	-	-	+	-	+	-	-	+	5	Atypical	TEST (-)	-
60	7 mo/M	-	-	-	-	Mild/right upper and lower limb	-	-	-	-	-	-	-	-	-	2	ILO	TEST (-)	TEST (-)
61	8 yr/F	-	-	+	-	Severe/ left upper and lower limb	-	-	-	-	-	-	-	-	-	4	Atypical	TEST (-)	-
62	7 mo/M	-	-	-	-	Mild/ right upper and lower limb	-	-	-	-	-	-	-	-	-	2	ILO	TEST (-)	-
63	2 mo/ M	-	-	-	-	Mild/ left upper limb	-	-	-	-	+	-	-	-	-	3	ILO	TEST (-)	-
64	2 mo/ M	-	-	+	-	Mild/ left upper and lower limb	-	-	-	-	+	-	-	-	-	5	Atypical	IC2-LoM	-
65	38/days/M	-	-	+	-	Mild/ left upper and lower limb	-	-	+	+	-	+	-	-	-	7	Classical	pUPD11	-
66	7 yr/ M	-	-	+	-	Mild/ left lower limb	-	-	-	-	+	-	-	+	-	6	Classical	IC2-LoM	-
67	15 mo/ M	-	-	+	-	Mild/ right upper and lower limb	-	-	-	-	-	-	-	-	+	5	Atypical	IC2-LoM	-
68	17 days/ M	-	-	+	-	Mild/ right upper and lower limb	-	-	-	-	-	-	+	-	-	5	Atypical	IC2-LoM	-
69	2 yr/ F	-	-	+	-	Severe/ left upper and lower limb	-	-	-	+	-	-	-	-	-	5	Atypical	pUPD11	-
70	5 mo/ F	-	-	+	-	Severe/right upper and lower limb	-	-	-	-	-	-	-	-	+	5	Atypical	IC2-LoM	-
71	2 mo/ M	-	-	+	+	Severe/right upper and lower limb	-	-	-	+	-	-	-	-	+	8	Classical	IC2-LoM	-
72	3 mo/ F	-	-	-	-	Mild/right upper and lower limb	-	-	-	-	+	-	-	-	+	4	Atypical	pUPD11	-
73	4mo /M	-	-	+	-	Mild/right lower limb	-	+	-	-	+	-	-	-	-	7	Classical	pUPD11	-
74	7 mo/M	-	-	+	-	Mild/right upper and lower limb	-	+	-	-	+	+	-	-	-	7	Classical	IC1-GoM	-

75	8 mo/ M	-	-	+	-	Mild/left upper and lower limb	-	+ B.	+	-	-	+	-	-	-	7	Classical	IC1-GoM	-
76	18 mo/ F	-	-	+	-	Mild/right upper and lower limb	-	-	-	-	-	+	-	-	-	5	Atypical	IC2-LoM	-
77	4,5 mo/ F	-	-	+	+	Severe/right upper and lower limb	-	-	-		+	-	-	-	-	7	Classical	IC2-LoM	-
78	18 mo/ F	-	-	+	-	Mild/left upper and lower limb	-	-	-	+	+	-	-	-	-	6	Classical	IC2-LoM	-
79	13 mo/ M	-	-	+	+	Mild/left lower limb	-	-	-	-	+	-	-	-	-	7	Classical	IC2-LoM	-
80	10 mo/ M	-	-	-	-	Severe/right lower limb	-	+	+	-	+	+		-	+	6	Atypical	pUPD11	-
81	7mo/ M	-	-	+	-	Severe/right lower limb	-	-	+	-	-	-	-	-	-	7	Atypical	pUPD11	-
82	4yr/F	-	-	+	+	Mild/right upper and lower limb	-	-	-	-	+	-	-	-	+	8	Classical	IC2-LoM	-
83	2 yr/M	-	-	+	+	Severe/right lower limb	-	-	+	-	-	-	-	-	+	6	Classical	IC2-LoM	-
84	4 mo/M	-	-	+	-	Severe/right upper and lower limb	+	-	-	+	-	+	-	-	-	8	Atypical	pUPD11	-
85	11mo/ M)	-	-	+	+	Severe/right upper and lower limb	-	-	-	-	-	+	-	-	+	8	Classical	TEST (-)	-
86	3 yr/M	-	-	-	-	Severe/right lower limb	-	+	-	-	-	-	-	-	-	3	ILO	TEST (-)	-
87	1 yr/M	-	+	-	-	Mild/right upper and lower limb	-	-	-	-	-	-	-	-	-	4	Atypical	TEST (-)	-
Total		**0**	**1**	**26**	**7**	**87**	**1**	**6**	**9**	**14**	**17**	**13**	**8**	**1**	**11**				

Abbreviations: M, Male; F,Female; mo, month; yr,year; B,Bening;WT: Wilms tumor; ILO: Isolated lateralized overgrowth; IC1-GoM, Imprinting Center 1 gain of methylation; ICR2-LoM, Imprinting Center 2 loss of methylation; pUPD11, Paternal uniparental disomy of chromosome 11. MS-MLPA: Methylation-specific multiplex ligation-dependent probe amplification
